# RB-TnSeq barcode abundance data sets for *Novosphingobium aromaticivorans* grown on the β-5-linked aromatic dimer dehydrodiconiferyl alcohol

**DOI:** 10.1128/mra.00844-24

**Published:** 2024-12-12

**Authors:** Fletcher Metz, Marco N. Allemann, Joshua K. Michener, Kevin S. Myers, Fachuang Lu, Daniel R. Noguera, Timothy J. Donohue

**Affiliations:** 1 DOE Great Lakes Bioenergy Research Center, University of Wisconsin, Madison, Wisconsin, USA; 2 Wisconsin Energy Institute, University of Wisconsin, Madison, Wisconsin, USA; 3 Laboratory of Genetics, University of Wisconsin, Madison, Wisconsin, USA; 4 Biosciences Division, Oak Ridge National Laboratory, Oak Ridge, Tennessee, USA; 5 Department of Civil and Environmental Engineering, University of Wisconsin, Madison, Wisconsin, USA; 6 Department of Bacteriology, University of Wisconsin, Madison, Wisconsin, USA; SUNY College of Environmental, Syracuse, New York, USA

**Keywords:** genomics, genetics, ligninolysis, aromatic compounds

## Abstract

A randomly barcoded transposon insertion sequencing (RB-TnSeq) library of *Novosphingobium aromaticivorans* DSM12444 was grown in media containing either glucose or the β-5-linked aromatic dimer dehydrodiconiferyl alcohol (DC-A) as the sole carbon source. The cultures were grown to saturation and then sequenced, yielding the barcode abundance data sets presented here.

## ANNOUNCEMENT

*Novosphingobium aromaticivorans* DSM12444 is an Alphaproteobacterium notable for its ability to metabolize various aromatic compounds (1–3) and funnel them into the production of commodity chemicals such as 2-pyrone-4,6-dicarboxylic acid (PDC) (3, 4) and *cis-cis-*muconic acid (5). These properties make this bacterium a promising chassis for microbial valorization of deconstructed lignin. A major barrier to lignin valorization is cleaving the variety of interunit linkages found between aromatic monomers so that the monomers can be funneled into products. *N. aromaticivorans* has been shown to catabolize aromatics joined by β-O-4 (6, 7), β−1 (8), and most recently, β-5 linkages (9). Dehydrodiconiferyl alcohol (DC-A) is a dimer composed of two aromatics joined by a β-5 (phenylcoumaran) linkage that has been used to study the catabolism of β-5-linked aromatics (10–14). To elucidate genes that are involved in DC-A catabolism in *N. aromaticivorans*, we used a randomly barcoded transposon insertion sequencing (RB-TnSeq) library to identify mutants that exhibited a fitness defect when grown using DC-A instead of glucose as a sole carbon source. Mutants with a fitness defect were defined as those with having a reduced abundance of associated barcodes after growing to saturation when utilizing DC-A as a carbon source compared to glucose (T0 condition). Barcode abundance data were collected as previously described (9) and briefly detailed below.

A previously generated RB-TnSeq library in wild-type *N. aromaticivorans* (15) was employed to screen for mutant fitness, using the barcode abundance data presented here (Table 1). An overnight culture of the library grown in lysogeny broth (LB) medium was diluted 1:100 into three flasks containing 2 g/L glucose in standard mineral base (SMB) minimal medium (7) and grown to saturation (~6.5 doublings, 14 hours). Each culture was then diluted to a starting cell density of 40 Klett units (~3.2 × 10^8^ colony forming units/mL) (7) with LB medium as a control or SMB minimal medium containing 1 g/L glucose or 1 g/L DC-A. The cultures were grown to saturation (~6.5 doublings, 9–39 hours, depending on carbon source), aliquoted, and stored at −80°C. The cells were later thawed and then harvested by centrifugation, resuspended in lysis buffer, and incubated at 65°C for 5 minutes. Genomic DNA was extracted using 25:24:1 phenol:chloroform:isoamyl alcohol (16). To prepare Illumina sequencing libraries, barcode DNA sequences were amplified from the genome using custom primers BarSeq_P1 and BarSeq_P2_IT001 to BarSeq_P2_IT009 (17) containing unique indices for each sample and Illumina adapter sequences. Barcode amplicons (378 bp average size with 20 bp barcodes) were quantified using a Qubit fluorometer and pooled before being sequenced at Azenta/GENEWIZ on an Illumina MiSeq with paired-end 150 bp reads (Illumina, San Diego, CA). De-multiplexing and quality control for the sequencing data were performed by bcl2fastq v2.20 using default parameters. Barcode frequencies and fitness values were calculated as previously described (17).

**TABLE 1:** Summary of RB-TnSeq sample data

Sample	Medium	Carbon source	Replicate number	Number of culture doublings	SRA accession number
RB-TnSeq T0 Glucose 1	SMB minimal medium	Glucose	1	6.36	SRX23809731
RB-TnSeq T0 Glucose 2	SMB minimal medium	Glucose	2	6.35	SRX23809732
RB-TnSeq T0 Glucose 3	SMB minimal medium	Glucose	3	6.34	SRX23809735
RB-TnSeq LB 1	LB medium	Glucose	1	6.98	SRX23809736
RB-TnSeq LB 2	LB medium	Glucose	2	6.98	SRX23809737
RB-TnSeq LB 3	LB medium	Glucose	3	6.95	SRX23809738
RB-TnSeq Glucose 1	SMB minimal medium	Glucose	1	6.41	SRX23809739
RB-TnSeq Glucose 2	SMB minimal medium	Glucose	2	6.41	SRX23809740
RB-TnSeq Glucose 3	SMB minimal medium	Glucose	3	6.38	SRX23809741
RB-TnSeq DC-A 1	SMB minimal medium	DC-A	1	6.96	SRX23809742
RB-TnSeq DC-A 2	SMB minimal medium	DC-A	2	6.95	SRX23809733
RB-TnSeq DC-A 3	SMB minimal medium	DC-A	3	6.98	SRX23809734

RB-TnSeq data sets have been crucial tools for the identification of metabolic pathways in *N. aromaticivorans* (8, 9, 15). Our RB-TnSeq data sets will enable investigators to identify the fitness effects of mutations in the genome of this bacterium when using DC-A as a carbon source.

## Data Availability

The raw RB-TnSeq reads are available from SRA as BioProject PRJNA1082384.
